# Optimised endoscopic access for intrajejunal levodopa application in idiopathic Parkinson's syndrome

**DOI:** 10.1007/s00702-023-02601-0

**Published:** 2023-02-21

**Authors:** K. E. Grund, A. Zipfel, B. Duckworth-Mothes, W. H. Jost

**Affiliations:** 1 Surgical and Experimental Endoscopy, University Hospital of Tübingen, Tübingen, Germany; 2 Working Group for Experimental Endoscopy, Education and Training, Department of General, Visceral and Transplantation Surgery, University Hospital of Tübingen, Tübingen, Germany; 3 Parkinson-Klinik Ortenau, Wolfach, Germany; 4 Present Address: Gottwollshaeuser Steige 25, 74523 Schwaebisch Hall, Germany

**Keywords:** Idopathic Parkinson syndrome, JET-PEG, Levodopa, Hybrid-PEG

## Abstract

Pump-guided intrajejunal levodopa administration is one of the indispensable forms of therapy in advanced Parkinson's syndrome, along with deep brain stimulation and subcutaneous apomorphine injection. The standard application of levodopa gel via a JET-PEG, i.e. a percutaneous endoscopic gastrostomy (PEG) with an inserted internal catheter into the jejunum, has not been unproblematic due to the restricted absorption area of the drug in the region of the flexura duodenojejunalis and especially due to the sometimes considerable accumulated complication rates of a JET-PEG. Causes of complications are mainly a non-optimal application technique of PEG and internal catheter as well as the often missing adequate follow-up care. This article presents the details of a—compared to the conventional technique—modified and optimised application technique, which has been clinically proven successfully for years. However, many details derived from anatomical, physiological, surgical and endoscopic aspects must be strictly observed during the application to reduce or avoid minor and major complications. Local infections and buried bumper syndrome cause particular problems. The relatively frequent dislocations of the internal catheter (which can ultimately be avoided by clip-fixing the catheter tip) also prove to be particularly troublesome. Finally, using the Hybrid technique, a new combination of an endoscopically controlled gastropexy with 3 sutures and subsequent central thread pull-through (TPT) of the PEG tube, the complication rate can be dramatically reduced and thus a decisive improvement achieved for patients. The aspects discussed here are highly relevant for all those involved in the therapy of advanced Parkinson's syndrome.

## Introduction

Levodopa is still the most important drug therapy for Parkinson's syndrome. However, experience shows that levodopa has to be administered more and more frequently during the course of the disease and that end-of-dose dyskinesias (hypo- and hyperkinesias) occur despite precisely timed oral dosing, forcing the use of alternative therapies (Salat and Tolosa 2013).

For such advanced stages of the disease, deep brain stimulation (DBS), subcutaneous apomorphine application by pump or intrajejunal levodopa application by pump are possible options (AWMF 2016).

The background for this therapeutically difficult situation is, in addition to the loss of dopaminergic neurons and the decrease in dopamine storage capacity (Jost et al. 2022), on the one hand the very short half-life/duration of action of levodopa and, on the other hand, the early involvement of the gastrointestinal tract in the symptoms of idiopathic Parkinson's syndrome (iPS) in the sense of a transit disorder. With subtle diagnostics, intestinal motility disorders are often detectable very early in the course of the disease (﻿Jost 2010). In addition to swallowing and transport problems in the oesophagus, gastroparesis, i.e. impaired motility of the stomach with slowed, uncoordinated or absent gastric emptying, is decisive, especially in advanced iPS (Warnecke et al. 2022). In the further course, this leads to a difficult, hardly or unpredictable transport of the drug into the duodenum or jejunum. Often the tablets get stuck in the oesophagus or do not reach their site of action in the proximal jejunum. This has correspondingly profound consequences for levodopa plasma levels in terms of fluctuations and pronounced ON/OFF problems.

**Fig. 1:**
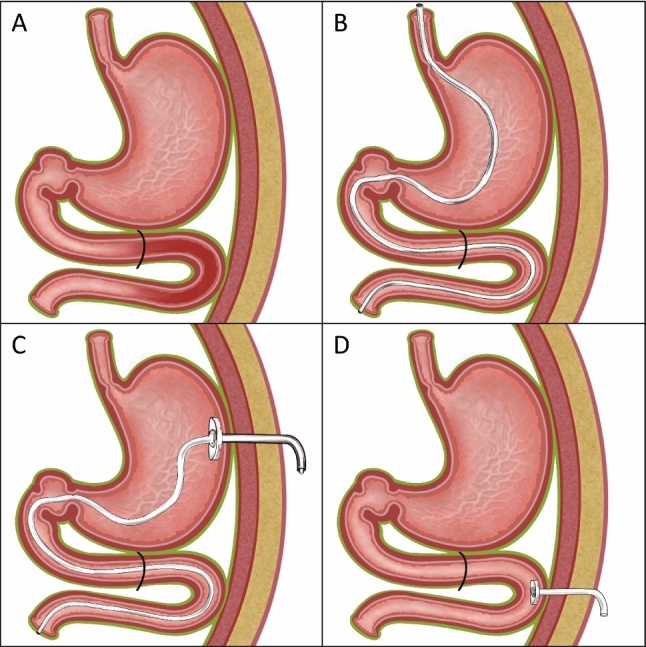
Possibilities of drug application into the proximal jejunum (red marking: access site). **A** Anatomical situation: restricted absorption area for levodopa: only in the first centimetres of the jejunum at the flexura duodenojejunalis. **B** transnasal (nasoenteric) tube, tip in target area. **C** JET-PEG: internal catheter through a PEG placed in the stomach, catheter tip in the target area. **D** EPJ = D-PEJ, direct puncture and probe insertion into the small intestine, inner retaining plate in the target area

Another fundamental problem of oral levodopa medication is the absorption of the active substance almost exclusively in the proximal jejunum. The local accuracy of the application (= topographical precision) is complicated by a restricted absorption area (10–20 cm) immediately after the flexura duodeno-jejunalis (see schematic drawing Fig. 1). Overview in (Warnecke et al. 2022).

This topographical factor—in addition to gastroparesis—makes oral therapy difficult to control and complicates gastral/jejunal administration of levodopa by tube.

Clinical experience shows that the problem awareness of the effects of gastroparesis and the limited absorption area should be deepened among neurological and gastroenterological colleagues.

In principle, the administration of drugs into the intestine is possible through a nasoenteric tube (NES) or through various percutaneous endoscopic gastrostomy (PEG) procedures (Fig. 1).

For the intrajejunal levodopa application discussed here—which is considered one of the three standard therapies for advanced Parkinson's syndrome—temporary (e.g. for testing levodopa administration), a nasoenteric tube can be inserted, but usually a PEG with an internal catheter, a so-called JET-PEG, is inserted for permanent therapy (Dam-Larsen et al. 2015; Lex et al. 2018; Gheorghe et al. 2019).

In principle, any PEG placement can be associated with complications (Grund and Zipfel 2016), which is why, on the one hand, acceptance is limited for some patients, and, on the other hand, high burdens arise for the patient and the healthcare system in the event of a complication. Interestingly, in recent LCIG studies, complications from the JET-PEG itself, tube and pump have been demonstrated in 60–100% of patients (Fernandez and Vanagunas 2013; Jost 2014; Nyholm and Stepien 2014).

In the relatively complex invasive procedure of a JET-PEG, problems should be avoided as far as possible from the outset to prevent complications by ensuring that all players have carefully and comprehensively undergone a hands-on training on the correct method in advance (Grund and Zipfel 2019).

The aim of this paper is to address the hitherto under-publicised problems of the conventional technique and to present and discuss the resulting alternatives.

The route of application via a JET-PEG, i.e. an internal catheter extending to the duodenal/jejunal region through a present PEG, which has so far been accepted as a given by the neurological side, must be discussed anew and critically from a surgical and endoscopic point of view—even now that there is an application procedure in the form of the Hybrid-PEG, which apparently involves considerably fewer complications than the conventional PEG techniques.

## Methods

Conventional delivery techniques as presented in the literature and in guidelines (Loser et al. 2005) have changed little in the 40 years since the inauguration of the method and have various weaknesses that have been shown to be the cause of the very high complication rates of PEG. In the literature, complication rates are usually only reported in a differentiated manner (Hucl and Spicak 2016; Boeykens and Duysburgh 2021), but in cumulation they are 40–75% (Rabast et al. 2008; Kratt et al. 2010; Schwartz and Ziegler 2022). These facts have led to the development of alternative techniques (Grund et al. 2006a; Grund and Zipfel 2016; Grund et al. 2021), which are based on (patho-) anatomical and (patho-) physiological facts as well as surgical considerations. With strict attention to the details described, this has led to a dramatic reduction in complication rates (cumulative complication rate dropped from 46 to 13%, early complications from 23 to 3%, late complications from 22 to 10% (Grund pers. comm. 2006)).

## Results/critical presentation of the conventional technique

As a result of the research in literature and guidelines as well as numerous hospitalizations and more than 100 PEG courses with several 1000 participants, it is clear that the conventional technique carries numerous possible sources of problems, which are presented here first:

### Transnasal tubes (TNT)

In the case of transnasal tube, frequent problems include relatively untargeted, inconsistently controlled tube insertion using the OTW/BTS technique, no targeted local anaesthesia but merely the application of anaesthetic lubricant to the tube, as well as incorrect ante- or reclination of the head during tube insertion. In principle, tube application beyond the flexura duodenojejunalis is also often difficult if the corresponding technical requirements are not respected (Grund and Ingenpaß 2007; Grund and Zipfel 2016).

### PEG in thread pull-through technique (TPT)

Complications during the insertion of a PEG in thread pull-through technique (TPT) are more frequent if the insertion is not prepared in a standardised manner (e.g. standardised instrument table), if diaphany and local anaesthesia are performed relatively untargeted and unsystematically in an aspiration technique, if the anaesthetic needle is not fixed in the gastric lumen, and if the insertion of the scalpel and thread trocar is performed without a support technique and without internal fixation.

Grasping the thread is often difficult in the gastric secretions, and there is a risk of losing the thread when it is pulled out of the mouth.

If the traction force is not well controlled when pulling the thread with the PEG tube through the abdominal wall, there is on the one hand the danger of injury to the base of the tongue by the thread and on the other hand the development of a pneumoperitoneum is programmed.

Serious complications are mainly triggered by the fact that the decisive adequate contact pressure until the peritoneal sheets are firmly connected (decisive safety factor in every PEG) is not controlled or not permanently guaranteed (Duckworth-Mothes et al. 2022).

### JET-PEG

Positioning the internal catheter in the optimal location in the flexura duodenojejunalis (Fig. 1) is often problematic due to technical problems with a normal gastroscope and insufficient BTS technique. Frequent dislocations of the internal catheter cause repeated follow-up interventions.

### Direct puncture

In this technique (better known as trocar puncture), 2 gastropexy sutures should be applied first (although 3 sutures are more favourable for topographical reasons alone). The subsequent puncture with the extremely pointed and sharp-edged trocar in the way specified in the instructions for use and literature carries the real risk of a faulty puncture of the posterior wall of the stomach, which can have life-threatening consequences due to the location of the aorta and pancreas in this area.

Simple measures can be taken to optimise the apposition area on the one hand and to eliminate the risk of malpuncture on the other (Grund et al. 2006a; Grund and Zipfel 2016; Duckworth-Mothes et al. 2022).

This method is not yet suitable for intrajejunal levodopa application via JET-PEG because the device does not allow for the insertion of a thinner internal catheter into the balloon catheter.

## Discussion/consequences for practice

The described deficiencies (see Chapter 3) of the conventional technique—in addition to problems with follow-up care (Farrag et al. 2019)—are undoubtedly to be regarded as the main causes of the high complication rates (Grund et al. 2006a, b; Grund and Ingenpass 2007). Recommendations for an alternative technique resulting from this deficit analysis have now been successfully applied to high numbers of patients (Grund et al. 2021; Kishta et al. 2021; Schumacher et al. 2022).

The decisive steps of these new developments are presented and discussed here, particularly with regard to the indication of levodopa application.

### Transnasal tubes, in this case nasoenteric tubes

In principle, such tubes are suitable for intrajejunal levodopa application (Fig. 1). However, their use for this indication is limited to a test phase (whether and how the local levodopa application works), since in the long-term course the inconvenience to the patient caused by the tube is usually not tolerated (Table 1).

**Table 1:** Advantages and disadvantages of nasoenteral feeding tubes

Nasoenteric feeding tubes	
Advantages	Disadvantages
Relatively simple placement of gastric tubes: "bed-side" method	Patient inconvenience, discomfort (quality of life $\Downarrow$)
Usually less invasive and minor risks during placement CAVE: Aspiration! Vagus reflex	Superficial mucosal lesions (erosions, ulcers, bleeding, pain …)
Easy to remove	Hygienic aspects (nose, mouth, throat, pharynx, sinus problems)
	Cosmetic disfigurement
	Swallowing problems
	Dislocation (quite common!)
	Tube occlusion, especially relatively frequent with thin tubes

### FDZ-PEG

Standardised and careful preparation of the patient, team and instruments are obligatory prerequisites after clear indication, information and consent of the patient ((Grund et al. 2006b) as well as Table 2).

**Table 2:** Preparations for PEG placement

PEG placement: obligatory requirements
Optimal preparation of the instrument table
Good insufflation
Clear diaphany
Accurate localisation and precise marking
Regular disinfection (large-scale, centrifugal, modo chirurgico)
Sterile drape (modo chirurgico)

It is generally important for the proper **technique** to be applied:

To reduce the complication rate, necessary technical details must be intensively brought to the attention of the doctor and assistant and carefully observed. This is indispensable for all types of PEG placement, but especially for critical cases, which undoubtedly include levodopa application in iPS. For these patients, a complication of any kind is particularly serious.

**Fig. 2:**
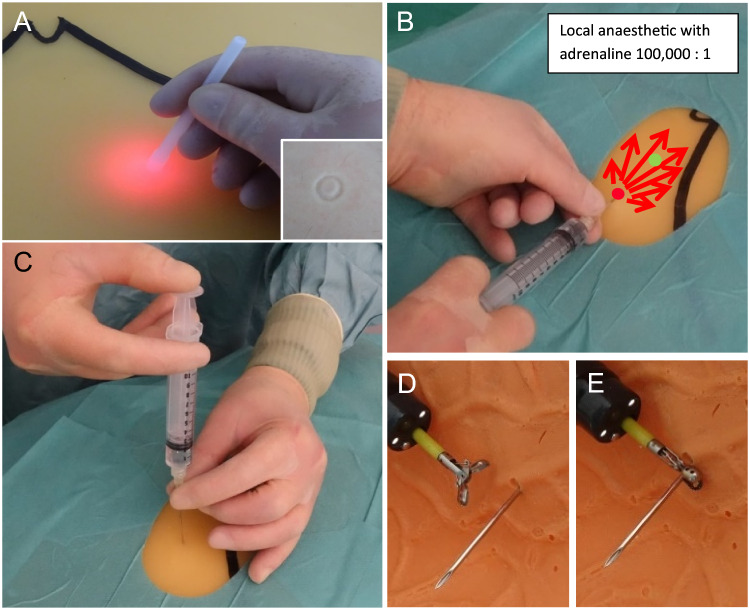
Initial steps for PEG placement. **A** Diaphany and marking. **B** Horizontal injection of local anaesthetic, superficial subcutaneous in a fan technique. Red dot: injection site of local anaesthetic, green dot: planned later insertion site of PEG. Note: hand position and support technique. **C** Vertical injection in support technique until the anaesthesia-needle has safely reached the stomach. **D** and **E** Here in the stomach the anaesthesia-needle is fixed close to the wall with endoscopic forceps

**Local anaesthesia** must be administered in a targeted and deliberate manner according to defined, layer-specific guidelines (Fig. 2).

For the following puncture, it is not sufficient to "… just poke it where it shines brightly", an exact localisation of the puncture site and a correct puncture direction must be ensured. If a JET-PEG is planned, the direction of the puncture should be slightly in the direction of the pylorus to prevent looping of the internal catheter.

The **aspiration test** (Ponsky 1998) often recommended in the literature to exclude intestinal interposition is uncertain in principle for several reasons (Grund et al. 2006b); in dubio, a sonographic check of the initial anatomical position should definitely be performed.

**Fig. 3:**
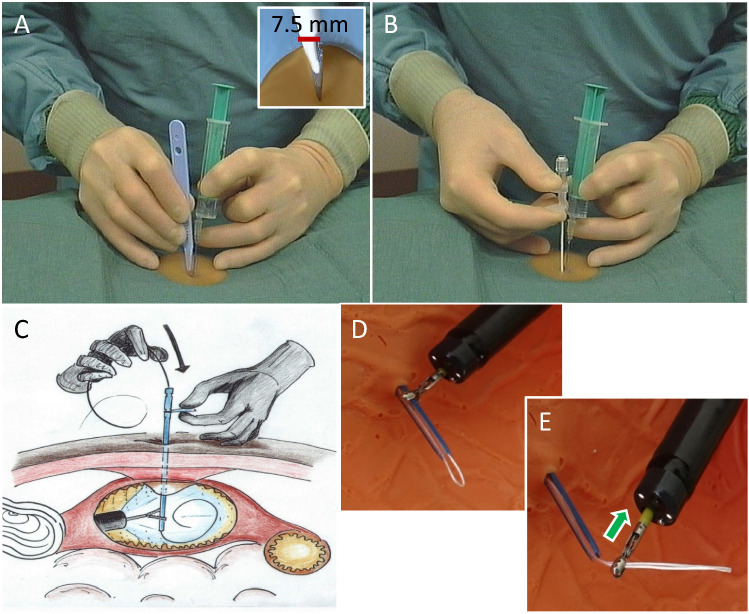
Scalpel incision and thread-trocar incision. **A** Converging puncture in support technique. Correct width of the stitch channel by No. 11 scalpel with 7.5 mm base width. **B** Insertion of the thread-trocar in identical manner, anaesthesia-needle and attached syringe serve as aiming aid. **C** Insertion of the thread. **D** and **E** Grasping the thread and securing it by pulling the forceps into the endoscope for a short distance

After insertion, immediate **fixation of the anaesthetic needle** in the stomach with endoscopic forceps is an essential detail to prevent complication-prone displacement of the wall layers against each other (Fig. 2). The trocar cannula should also be fixed internally until the suture is inserted into the stomach (Fig. 3 D).

The **puncture incision** (obligatory for several reasons with a No.11 scalpel, see Fig. 3A) and insertion of the suture trocar in a support technique follow the laws of trigonometry (Fig. 3A, B).

**Fig. 4:**
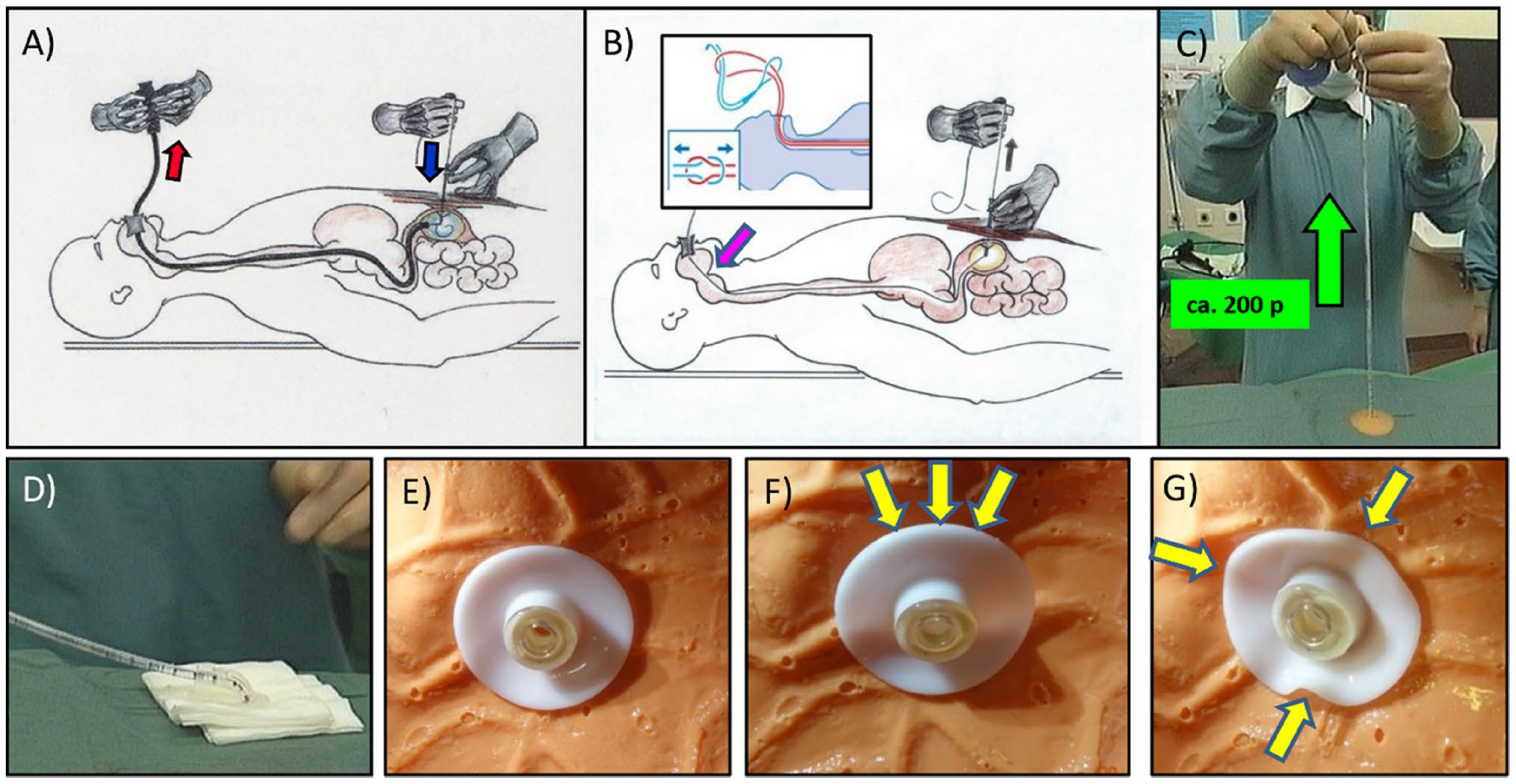
Further steps until the PEG tube is passed through the abdominal wall. **A** Endoscope together with forceps and thread is withdrawn orally. **B** The thread-loop and the PEG tube are connected in front of the mouth, then the tube is pulled outwards through the abdominal wall by the abdominal thread using short-stroke wrapping movements. Protect the base of the tongue with your finger. **C** Immediately after the tube has passed through, it is held under slight tension (prophylaxis of a pneumoperitoneum, thereby increasing diagnostic certainty in the event of the occurrence of abdominal pain after the PEG: If, despite this pneumoperitoneum prophylaxis, a pneumoperitoneum can be detected postinterventionally, this indicates a complication that should be taken seriously and often needs for surgical intervention). **D** Fixation of the external retaining plate under the same defined traction and dressing. **E** Correctly retracted retaining plate; it lies smoothly against the stomach wall. **F** Retaining plate too loose, risk of peritonitis and infection. **G** Retaining plate too tight, risk of buried bumper syndrome (BBS)

When **passing** the suture and the PEG tube from intragastral to external (Fig. 4), the base of the tongue must be protected with the finger to avoid bleeding (Fig. 4B).

There is a simple, easy-to-perform prophylaxis for easily **avoiding pneumoperitoneum** and thus diagnostic uncertainty (Fig. 4C).

The tightening force should be approx. 200 p (weight of 2 bars of chocolate) (Grund et al. 2006b) and neither fall below (risk of peritonitis and infection) nor exceed (risk of buried bumper syndrome (BBS)) (Table 3).

**Table 3:** The 14 steps for TPT-PEG.

TPT PEG step by step
1. Orientating gastroscopy deep into the duodenum, possibly even into the jejunum
2. Good insufflation of the stomach
3. Search for diaphany, finger pressure test and marking
4. Local anaesthesia: a) horizontal in fan technique, b) vertical to the stomach
5. Fixation of the anaesthesia-needle close to the wall in the gastric lumen using forceps
6. Puncture incision with the No. 11 scalpel using the convergence technique
7. Insertion of the thread trocar in the scalpel channel
8. Removal of the steel cannula, fixation of the trocar sleeve in the gastric lumen (see 5.)
9. Insertion of the thread, grasping with the forceps and a few cm of retraction into the endoscope
10. Extraction of endoscope and thread orally
11. Connecting the thread and PEG tube in front of the mouth
12. Tongue protection with the finger
13. Pulling the thread and PEG tube through the abdominal wall by short-stroke wrapping movements
14. Fixation of the tube under defined tension (approx. 200 p), dressing

### HYBRID PEG

The technique of thread pull-through PEG (FDZ-PEG) just described can be decisively improved by combining it with gastropexy (endoscopic suturing of the anterior gastric wall to the abdominal wall) (Hybrid-PEG) (Grund et al. 2021; Kishta et al. 2021; Duckworth-Mothes et al. 2022).

**Fig. 5:**
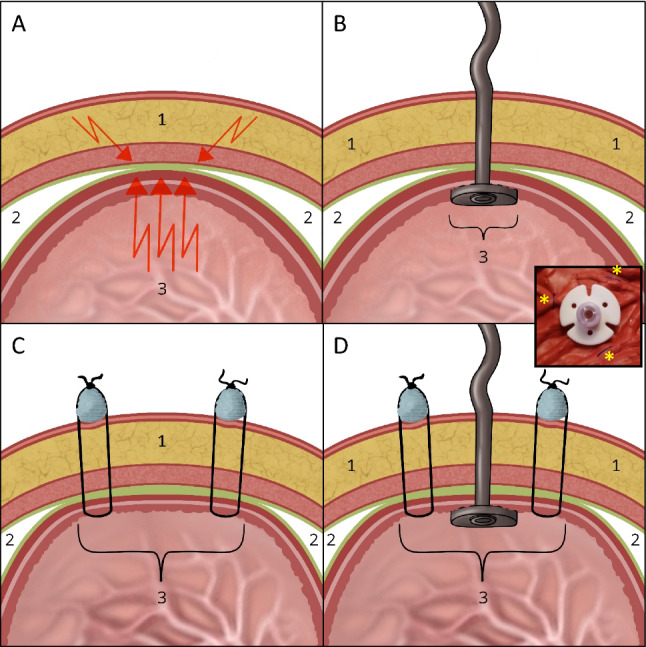
Decisive local situation for the safety of a PEG. **A** It is well known that the stomach and the anterior abdominal wall must remain fixed in direct contact for many hours so that first fibrin adhesion and then connective tissue organisation can take place (red arrows) 1: Abdominal wall 2: Free abdominal cavity 3: Stomach **B** With a normal TPT: this is only possible directly under the holding plate (3) and with a prolonged optimal pressure force, which is difficult to guarantee in practice. **C** The solution to the problem lies in a primary endoscopically controlled gastropexy with three sutures. This results in a large contact area and optimal safety due to a large-area with immediate and also permanent apposition of the serosa layers (3). **D** Hybrid PEG: A normal TPT PEG is placed in the centre of the three gastropexy sutures, which can be loosely attached to the stomach wall here. Peritonitis, infection or BBS are safely avoided. Inset: Inner retaining plate with the three gastropexy sutures (yellow asterisks)

In general, the decisive factor for low complication rate of a PEG is the secure and immediate adhesion as well as permanent adhesion and sealing of the two serosa surfaces (peritoneum of the anterior abdominal wall and outer surface of the stomach) (Fig. 5).

The combination of a primary gastropexy with subsequent TPT PEG in the centre of the 3 obligatory pexy sutures in the form of a Hybrid-PEG (Fig. 5) shows very good results with significantly lower complication rates than with conventional techniques (Kishta et al. 2021; Schumacher et al. 2022; Bojarski pers. comm.). Even if direct head-to-head RC-studies (which may be ethically problematic regarding the clear benefits of Hybrid-PEG) are missing, the plausibility of the method and the cited clinical results are convincing.

Hybrid PEG certainly has the potential to become the future standard for PEG systems (Kishta et al. 2021).

**Fig. 6:**
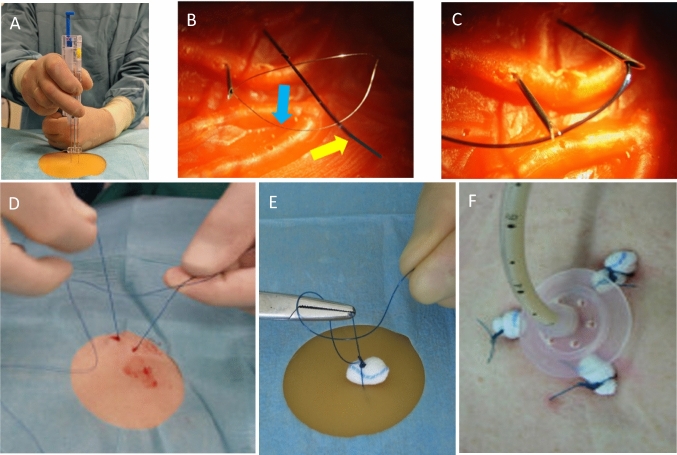
Details for gastropexy: **A** Insertion of the gastropexy device under support technique. **B** after the double needle appears in the lumen, the catcher snare is advanced (blue arrow) and the thread from the second needle is inserted through the catcher snare (yellow arrow). **C** by closing the catcher snare, the thread is clamped at the needle. **D** after removing the suturing device, the sutures appearing on the abdominal wall can be knotted. **E** an instrumental knot is recommended for this (3 double knots in opposite directions, a mini ball swab ("Fips") is included under the knot. **F** After completion of 3 (!!!) gastropexy sutures, an TPT is placed in the centre, during which the retaining plates can be adapted loosely

Few but crucial details apply to the placement of gastropexy sutures (Fig. 6, Table 4). The important ones are (Grund et al. 2006a; Grund et al. 2021; Duckworth-Mothes et al. 2022):

- Control and **correct handling** of the pexy device with regular application of at least 3 sutures.
- Adequate **knotting technique** for safe apposition of the wall layers (6 knots in opposite directions).
- Consistent use of **mini ball swabs** (so-called "fipses") to underlay the knots to protect the skin, reduce pain and facilitate suture removal. (Fig. 6E, F).

**Table 4:** 12 steps of gastropexy

Gastropexy step by step
1. Careful inspection of the gastropexy suturing apparatus in all functions
2. Preloading the thread and adjusting the slide
3. Particularly extensive local anaesthesia, especially horizontally
4. Inserting the double needle into the stomach
**5.** Advancing the catcher snare
6. Inserting the thread through the catcher snare
7. Closing the catcher snare and belaying (light counter-holding on the safety sling)
8. Pull out the suturing device completely and
9. secure the two thread ends immediately
10. Knot the threads securely, place mini-swab underneath beforehand
11. THREE such sutures are applied
12. Breathe a sigh of relief

### JET-PEG

**Fig. 7:**
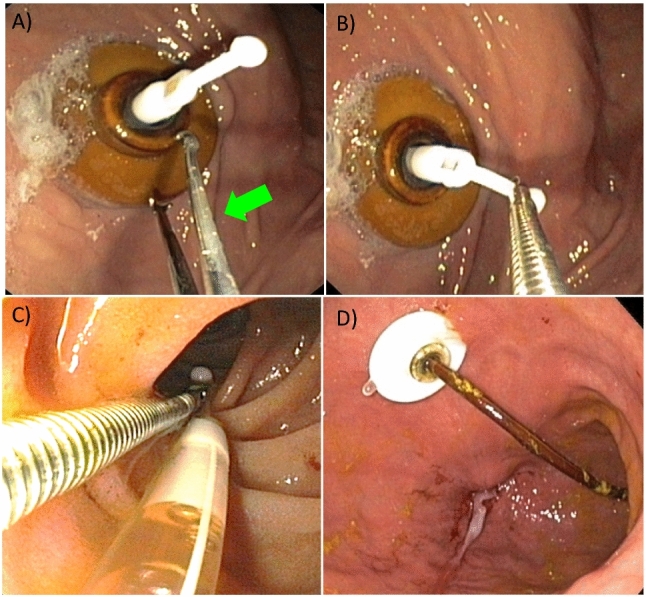
JET-PEG: an internal catheter is advanced through the PEG and inserted into the proximal jejunum. **A** Inner catheter tip appears in the gastric lumen (green arrow: prepared grasping forceps). **B** Tube tip grasped. **C** The inner catheter is brought into the proximal jejunum using the dragging technique (BTS). **D** Optimally positioned inner catheter

**Fig. 8:**
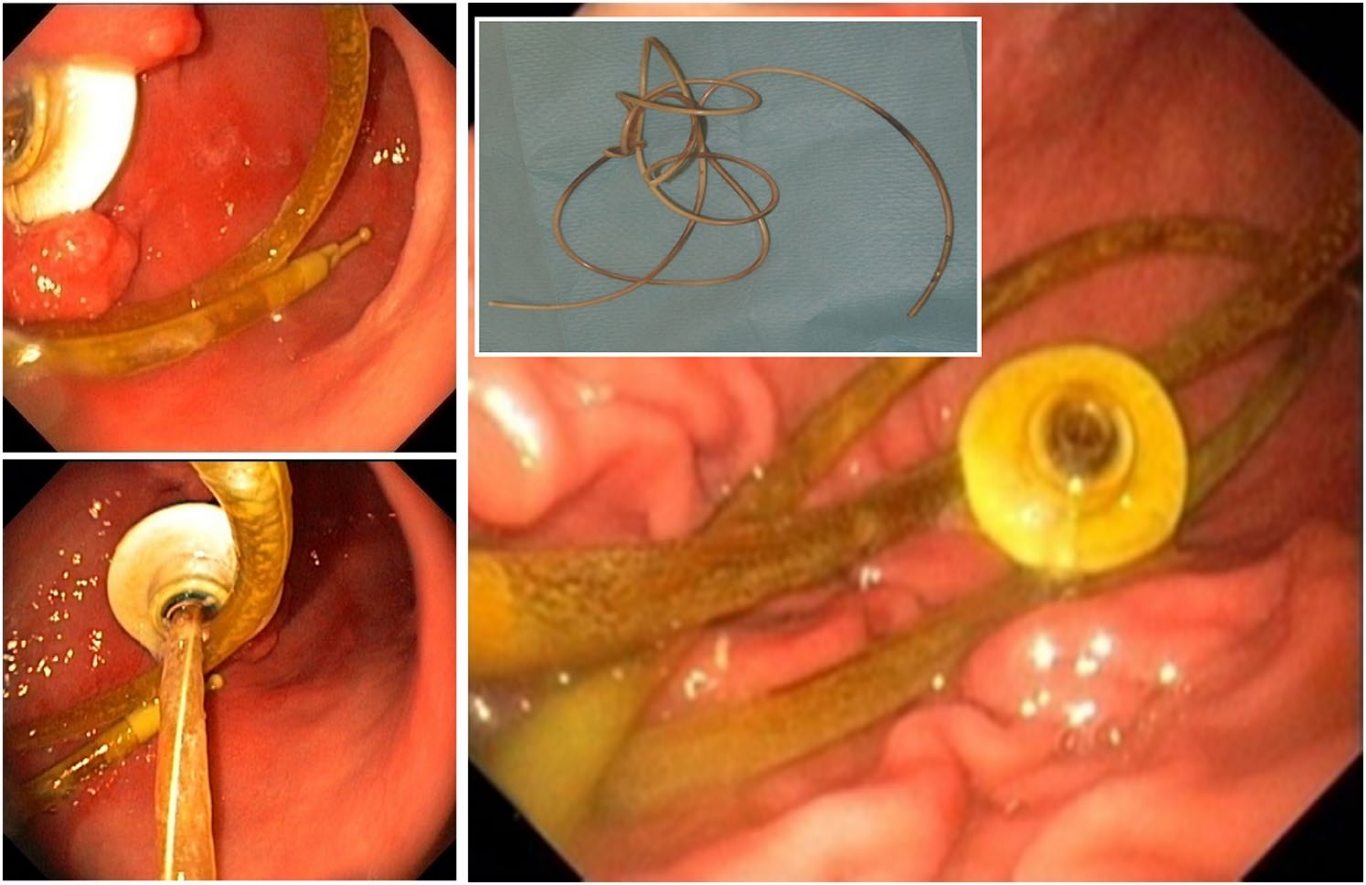
Common complications after placement of the internal catheter: dislocation into the stomach, knotting, dislocation due to loss of external connectivity

The placement of an inner catheter into a PEG (so-called JET-PEG) (Fig. 7) has been associated with a high rate of dislocations (Fig. 8, Table 5), which is particularly undesirable for the indication "levodopa application".

**Table 5:** Problems of JET-PEG

JET-PEG: main problems
Placement of inner catheter often difficult
Unfavourable external conditions (intensive care unit, intensive care patient, Friday evening, no competent assistance)
Gastroscope "too short", paediatric colonoscope missing
Little space for "manoeuvring"
No suitable long forceps available
Lack of expertise of endoscopist and/or assistant
Dislocation already during retraction of the endoscope
Checking the position of the tube (X-ray!): Logistics problem
Professional after-care: not available
Frequent: inner catheter dislocation in the course is often only noticed late (too late)

**Fig. 9:**
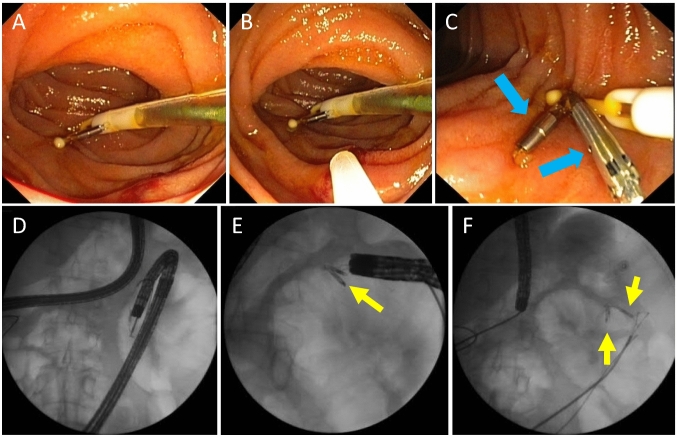
In case of recurrent dislocation, prophylactic clip fixation of the inner catheter tip to the jejunal wall. **A**–**C**: Fixation with 2 haemostatic clips. **D**–**F**: radiological control of fixation exactly in the target area at the first jejunal loop, close to the flexura duodenojejunalis (see **D**). Yellow/blue arrows: Clips

Primary and secondary dislocation can often be avoided by:

- For primary insertion, direction of insertion towards the pylorus.
- Safe mastery of **OTW and BTS techniques** (Grund and Ingenpaß 2007) for correct positioning of the inner catheter tip.
- Avoiding tube portions that are too short or too long in the stomach.
- Avoiding tube disjunction in the area of the Y-piece.
- Fixation of the tube tip to the intestinal wall using haemostatic clips in case of recurrent dislocation (Fig. 9).

### Direct puncture = trocar puncture

The puncture of the extremely sharp, high-calibre steel trocar requires special technical precautions (Grund et al. 2006a):

To avoid serious complications, it is essential to observe:

- During the puncture, a strong counter-traction is required upwards on the 3 pexy-sutures left longer.
- A correct pulsed puncture technique under obligatory endoscopic view is essential.

### EPJ = D-PEJ

**Fig. 10:**
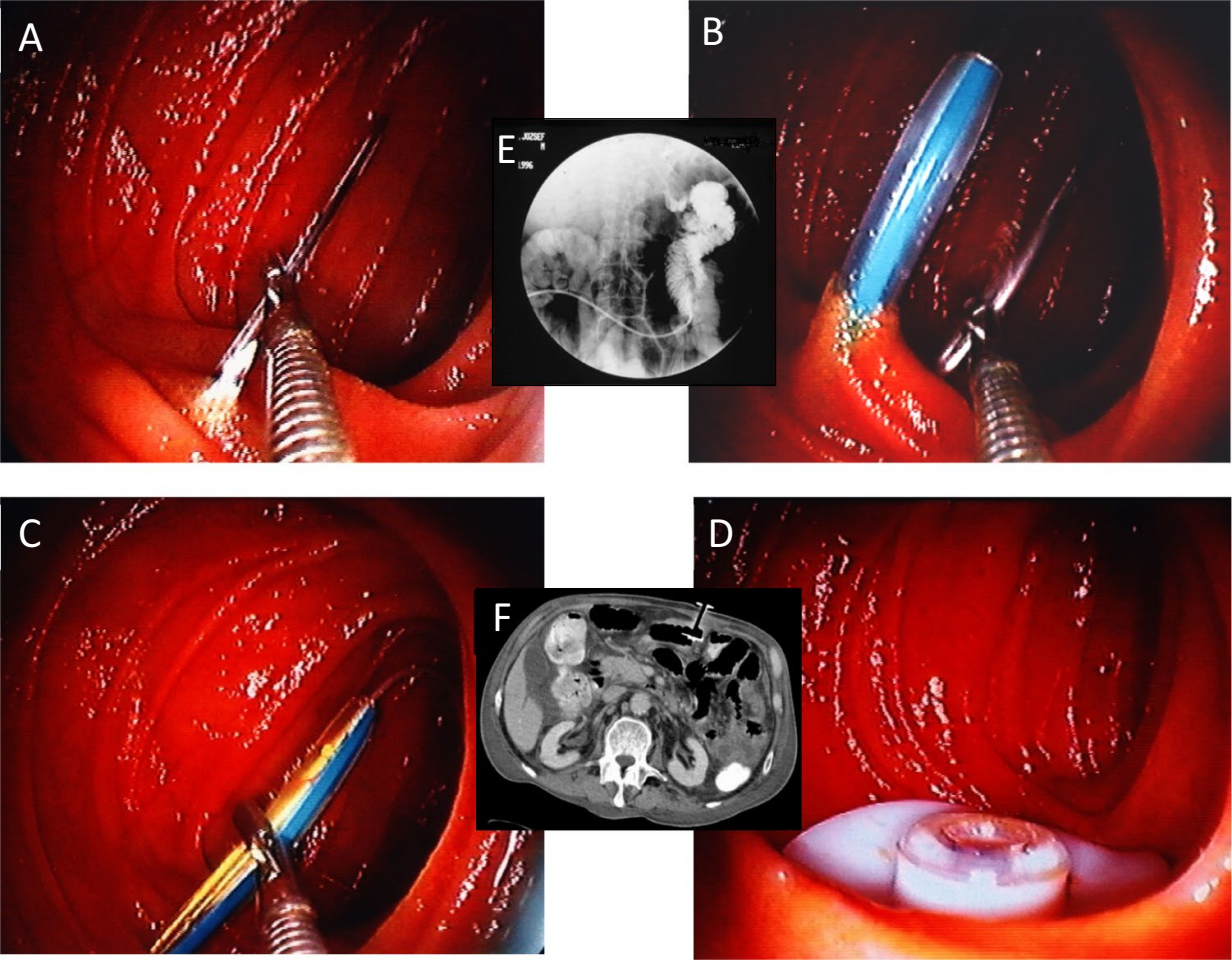
EPJ = D-PEJ; here the small intestine is punctured directly using the FDZ technique, thus creating direct access to the jejunum. **A** Fixation of the anaesthesia-needle. **B** Parallel insertion of the trocar (blue). **C** Internal fixation of the trocar and then grasping of the inserted thread. **D** Internal retaining plate in ideal position on the small intestine wall. **E** radiological control with contrast medium: EPJ lies correctly in the first jejunal loop. **F** CT check: correct position

Direct puncture of the small intestine is a recent development of PEG techniques (Fig. 10) (Mellert et al. 1994; Grund et al. 2006b) and is relevant as a substitute for a PEG in the case of a stomach that is no longer present or inaccessible in the surgical and intensive care context. The technique is quite critical and requires a decided and standardised procedure as an absolute prerequisite for similar success rates as with "normal" PEG. Defined prerequisites are (Grund et al. 2006a; Duckworth-Mothes et al. 2022):

- Very exact diaphany and precise marking of the puncture site.
- Obligatory application of spasmolytic drugs.
- Internal fixation of anaesthetic needle and suture trocar without exception.
- Particularly careful thread passage.

In principle, an EPJ/D-PEJ would be ideal as a form of administration for intrajejunal levodopa administration, as the first jejunal loop is usually punctured just behind the flexura duodenojejunalis and thus the ideal resorption site for levodopa can largely be optimally hit.

However, the relatively demanding puncture technique, which requires a very experienced endoscopist, as well as concerns about complications with an TPT-only technique at this site (see above) have so far prevented larger numbers of PD patients from receiving an EPJ.

Newly developed puncture devices under development/trial could bring about a change here and establish the EPJ/D-PEJ in Hybrid technique as the optimal form of application for levodopa intrajejunal.

Another advantage of the EPJ technique would be the very short distance that the levodopa gel has to pass in the relatively narrow tube lumen (a few centimetres from the abdominal surface to the jejunum in contrast to > 60 cm with a JET-PEG) (see “Hydrodynamics of tubing systems”).

### Hydrodynamics of tubing systems

So far, (too) little attention has been paid to the physics of flow in (narrow) tubes for levodopa application. Here, Hagen–Poiseuille's law (1839) plays an important role. It states that the inner diameter of the tube, which affects the 4th power, is the decisive factor for the volume flow or the required pressure. This has considerable consequences for small-calibre tube systems when applying the relatively highly viscous LCIG/LECIG gel into the intestinal lumen and represents a relatively frequent source of complications. These physical facts should also be given more attention in the context of medical technology.

### Pumps

Pumps also appear to be frequent sources of complications (personal communication). Recent developments in mechatronics and accumulator technology (LiPo, LiFePo) have brought significant advances in the field of technology for portable pumps through increasing miniaturisation and extended running times, which above all makes outpatient care much easier. Unfortunately, the connecting pieces of the tubes are still different and incompatible in some cases.

## Summary

Intrajejunal pump-controlled administration of levodopa is a standard form of therapy for advanced Parkinson's syndrome. It is realised via a so-called JET-PEG, i.e. a PEG with an inserted internal catheter into the jejunum.

In view of the high complication rates, a critical analysis reveals an urgent need for improvement of the JET-PEG, especially by optimising the insertion technique. To achieve this, numerous modifications and optimisations of the conventional technique and strict adherence to the developed technical details are goal-oriented. In addition, new developments such as the Hybrid-PEG (combination of 3 gastropexy sutures with subsequent central thread pull-through technique of the PEG tube) contribute to a dramatic reduction in the complication rate. Further developments, especially with regard to application devices, are needed to make intrajejunal levodopa application even safer and more effective for the critical patient group with advanced iPS.

## Considerations for practice

All physicians and assistants who care for patients with advanced iPS should be informed about the possibilities and problems of intrajejunal targeted levodopa application and work together to ensure that the multiple possibilities of avoiding complications can be realised through an optimised application technique when applying a JET-PEG. In principle, such patients should be treated in centres with appropriate experience and optimal follow-up care should be ensured.

## Data Availability

The datasets generated during and/or analysed during the current study are available from the corresponding author on reasonable request.

## Abbreviations

BBS: Buried bumper syndrome
BTS: Beneath the scope
DBS: Deep brain stimulation
D-PEJ: Direct percutaneous endoscopic jejunostomy = EPJ; Note: D-PEJ and EPJ are different names for the same procedure.
EPJ: Endoscopic percutaneous jejunostomy = D-PEJ
TPT: Thread pull-through
IPS: Idiopathic Parkinson's syndrome
JET-PEG: Jejunal tube through PEG
LCIG: Levodopa-carbidopa gastrointestinal gel
NES: Nasoenteric tube
OTW: Over the wire
PEG: Percutaneous endoscopic gastrostomy
TNT: Transnasal tube
